# Parkinson's Disease and Legal Capacity

**DOI:** 10.1002/alz70857_096425

**Published:** 2025-12-24

**Authors:** Panagiota Voskou

**Affiliations:** ^1^ University of Athens, Athens, ‐, Greece

## Abstract

**Background:**

One recent area of study in Parkinson's Disease (PD) concerns financial capacity and medical decision‐making capacity. Patients frequently make treatment and research participation decisions. Obtaining informed consent to treatment is an important medical‐legal and clinical aspect of neurological practice.

**Method:**

Pubmed database has been used, by keywords: Parkinson's disease; legal capacity; competency

**Result:**

Regarding treatment decisions, PD patients with dementia (PDD) have been found impaired on three clinical standards: understanding; reasoning; appreciation. They showed better understanding of the treatment situation relatively to mild Alzheimer disease patients, but more difficulty for simple choices. Financial incapacity was also found in PDD. Due to executive dysfunction, a major neurocognitive factor for incompetency in PD, even patients with cognitive impairment without dementia show impaired ability to make treatment choices through deficits in encoding, organization of new medical information and not reasoning or appreciating that information. Furthermore, depression, which affects cognitive functions, is common in PD. This further complicates the assessment of capacities. In fact, PD patients’ cognitive functioning and financial capacity can be negatively influenced by apathy more than depression.

**Conclusion:**

There are limited studies regarding legal capacity assessment in PD, since deficits in capacities are often related to progressive motor dysfunction. There is need for studies regarding the relationship of specific brain areas linked to apathy and components of legal capacities in PD. Inquiries about the patient's wishes early in the course of PD, before cognitive impairment arises, are also recommended. Finally, clinicians and researchers should carefully assess decisional capacity in PD patients with cognitive impairment.

